# The Connection Between Deception Detection and Financial Exploitation of Older (vs. Young) Adults

**DOI:** 10.1177/07334648211049716

**Published:** 2021-10-12

**Authors:** Christopher A. Gunderson, Leanne ten Brinke

**Affiliations:** 1 2927 University of Denver, Denver, CO, USA; 2 97950 University of British Columbia Okanagan, Kelowna, BC, Canada

**Keywords:** fraud, deception detection, social cognition

## Abstract

Although poor deception detection accuracy is thought to be an important risk factor for fraud among older adults, this link has not been explicitly studied. Using a cross-sectional design, older and young adults viewed and made judgments of real, high-stakes truths and lies with financial consequences. Older (vs. young) adults exhibited a greater truth bias when evaluating individuals pleading for help in finding a missing relative, which was associated with greater donations to deceptive pleaders. However, all participants were highly vulnerable to fraud. Future research should consider both risk and protective factors affecting financial fraud across the lifespan.

Fraud is the most common form of elder abuse and costs older adults in the U.S. upwards of $36 billion each year (TrueLink, 2019). Some instances of fraud involve a breach of trust between an older individual and a family member or close friend who misuses funds. Other instances—namely, fraud—involve manipulation by strangers. Older adults sometimes willingly provide their funds to nefarious actors in response to deceptive pleas for help or disreputable advice (Weissberger et al., 2019). Accordingly, older (vs. young) adults’ ability to detect deception is thought to play an important role in their vulnerability to fraud (Spreng et al., 2021).

The propensity to trust others appears to increase across the lifespan (Poulin & Haase, 2015). Older (vs. young) adults report significantly higher levels of generalized trust toward family, friends, neighbors, and strangers (Li & Fung, 2012). Increased trust among older adults also appears in the context of deception detection: older (vs. young) adults are more likely to mislabel liars as truth-tellers—suggesting a truth bias in their judgments (Ruffman et al., 2012; Stanley & Blanchard-Fields, 2008).

A truth bias may increase compliance with requests for money or assistance, making older adults vulnerable to fraud. Here, we present a novel paradigm for directly examining the connection between deception detection and fraud. We examine older (vs. young) adults’ ability to discriminate liars from truth-tellers in real, high-stakes appeals for help. Further, we consider the sensitivity and bias of these judgments to determine how these performance metrics affect the risk of fraud.

## Method

### Participants

One-hundred and nineteen participants were recruited for the study. We recruited 61 young adults (48 women, 13 men; *M*_*age*_ = 19.03, *SD* = 1.47, and range = 18–27) from the University of Denver. The young adult sample was primarily Caucasian (75.4% White, 13.1% Asian American, 8.2% Latino/a, 1.6% African American, and 1.6% Middle Eastern). Fifty-eight older adults were recruited (43 women, 15 men; *M*_*age*_ = 74.12, *SD* = 8.21, and range = 60–93) from retirement communities and a continuing education organization for older adults in the Denver area. The older adult sample was primarily Caucasian and less diverse than the young adult sample (98.3% White and 1.7% African American). All participants reported normal or corrected-to-normal vision and hearing. Sensitivity power analyses (1− β = .80; α = .05) indicated that this sample size was sufficient to detect a medium effect size (*d* = .52) on independent samples *t-*tests, and a medium-sized interaction (Cohen’s *f* = .26) in a 2 × 2 mixed ANOVA. This research was reviewed and approved by the Institutional Review Board at the University of Denver (Protocol No. 989739-17).

### Materials

#### Emotional pleas

Participants viewed 12 individuals pleading for the return of a missing relative. Videos were chosen from ten Brinke and Porter’s (2012) sample of *N* = 78 using stratified random sampling to ensure an equal number of men and women, genuine and deceptive pleaders. *Deceptive pleader* videos depicted individuals who were later convicted of murdering the missing relative based on strong physical evidence (e.g., DNA, possession of the murder weapon). *Genuine pleaders* were not involved in the disappearance of the missing relative; the missing individual was either found in the absence of foul-play, or another individual was convicted of their murder based on strong physical evidence. Participants watched six genuine (three men and three women) and six deceptive (three men and three women) pleaders. On average, videos were 35.42 seconds long (*SD* = 40.38).

### Procedure

Participants provided consent and older adults completed a consent “quiz” to ensure they understood the information presented in the consent form. Participants also completed the Mini-Cog assessment of cognitive impairment (Borson et al., 2003): older adults (*M* = 4.33, *SD* = .78) did not differ from young adults (*M* =4.31, *SD* = 1.13), *t*(116) = −.06, *p* = .95, *d* = −.07. Participants then watched the emotional pleas in random order. After each emotional plea, participants were asked to judge the pleader’s veracity (lying or telling the truth). To measure financial consequences, participants indicated how much (hypothetical) money ($0–$100) they would donate to a GoFundMe fundraiser supporting the search for each missing person. Finally, participants completed a series of surveys and demographic questions.^1^

### Data Preparation

Accuracy was calculated for each participant as a percentage of all 12 videos that were correctly labeled as liars or truth-tellers. Accuracy (%) was also calculated for genuine and deceptive videos, separately. Poor accuracy could result from either poor sensitivity to cues to deception, a decision-making bias, or both. To avoid confounding these factors, we also used signal detection analysis (Stanislaw & Todorov, 1999) to calculate *sensitivity* (*d’*)—the ability to discriminate between truths and lies—and *criterion* (*c*)— the tendency to favor a particular response (e.g., a truth judgment). We first calculated the *hit rate* (correctly identifying a liar) and the *false alarm rate* (misidentifying a truth-teller as a liar) and replaced extreme values of 1 or 0 with .99 and .01, respectively (Macmillan & Kaplan, 1985). Hits and false alarms were then standardized, and sensitivity was calculated by subtracting standardized false alarms from standardized hits. Sensitivity indexes observers’ ability to discriminate lies from truths, where positive scores indicate good discrimination accuracy, negative scores indicate mislabeling of liars as truth-tellers (and vice versa), and scores near zero indicate a lack of discrimination. Criterion was calculated by adding the standardized hit rate to the standardized false alarm rate and multiplying by −.5. Criterion indexes observers’ response bias, where positive scores indicate a truth bias, negative scores indicate a lie bias, and scores near zero indicate no response bias.

## Results

### Deception Detection Performance

#### Sensitivity and bias

Older (*M* = .60, *SD* = .91) and young (*M* = .40, *SD* = 1.00) adults did not differ in their sensitivity to deception, *t*(117) = 1.15, *p* = .253, *d* = .21. However, sensitivity across both age groups (*M* = .50, *SD* = .96) was significantly greater than 0, *t*(118) = 5.65, *p* < .001, *d* = .52, indicating an ability to discriminate between truth-tellers and liars. With respect to criterion, older adults (*M* = .44, *SD* = .75) exhibited a significantly greater truth bias than young adults (*M* = .12, *SD* = .42), *t*(117) = 2.82, *p* = .006, *d* = .52. However, both older, *t*(57) = 4.42, *p* < .001, *d* = .75, and young adults’, *t*(61) = 2.26, *p* = .027, *d* = .42, mean criterion was greater than 0—indicating a truth bias. See Table 1 for correlations between all variables.

**Table 1.** Pearson Correlations Between Age, Participant Gender, Mini-Cog Scores, Accuracy (%), Sensitivity (*d’)*, Criterion (*c*), and Financial Donations to Genuine and Deceptive Pleaders.

	1	2	3	4	5	6	7	8	9
1. Age (continuous)	—								
2. Gender	−.05	—							
3. Mini-Cog total	−.06	.08	—						
4. Accuracy –									
Genuine pleaders (%)	.20^*^	.02	.00	—					
5. Accuracy –									
Deceptive pleaders (%)	−.09	−.09	.09	−.20^*^	—				
6. Sensitivity (*d’*)	.09	−.05	.12	.55^**^	.66^**^	—			
7. Criterion (*c*)	.24^**^	.08	−.05	.69^**^	−.79^**^	−.15	—		
8. Donations ($) to genuine pleaders	−.07	.15	.06	.21^*^	−.01	.16	.09	—	
9. Donations ($) to deceptive pleaders	−.09	.20^*^	.01	.03	−.33^**^	−24^**^	.21^*^	.82^**^	—

*Note. N* = 119; gender: female = 0, male = 1. *p < .05; **p < .001.

#### Accuracy (%)

Across all participants, overall accuracy (*M* = .57, *SD* = .13) was significantly above chance (i.e., .50), *t*(118) = 5.73, *p* < .001, 95% CI [.55, .59], *d* = .53, 95% CI [.33, .72]. While truth accuracy (*M* = .66, *SD* = .19) was significantly above chance, *t*(118) = 9.17, *p* < .001, 95% CI [.62, .69], *d* = .84, 95% CI [.63, 1.05], lie accuracy (*M* = .49, *SD* = .23) did not differ from chance, *t*(118) = −.73, *p* = .47, 95% CI [.44, .53], *d* = −.07, 95% CI [-.25, .11].

#### Fraud vulnerability

Descriptively, 95% of our sample gave a hypothetical donation to genuine pleaders, whereas 93.3% of our sample donated to deceptive pleaders. Concerning the amount of those donations, a 2 (veracity: genuine v. deceptive) × 2 (age group: older v. young adults) mixed ANOVA with hypothetical donations to pleaders as the dependent variable indicated a significant main effect of veracity, *F*(1, 117) = 46.32, *p* < .001, η_p_^2^ = .28. Participants indicated that they would donate more money to genuine (*M* = 41.22, *SD* = 25.86), relative to deceptive (*M* = 31.59, *SD* = 25.41), pleaders. However, there was no significant main effect of age, *F*(1, 117) = .70, *p* = .406, η_p_^2^ = .006, nor an age × veracity interaction, *F*(1, 117) = .58, *p* = .447, η_p_^2^ = .005.

**Figure 1.**
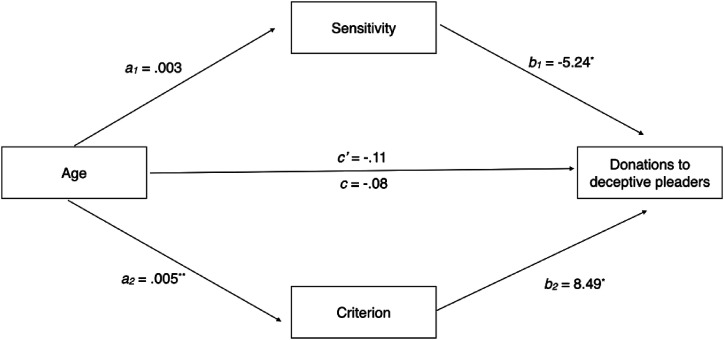
Multiple mediation of the effect of age (young adult = 0 and older adult = 1) on donations to deceptive pleaders through sensitivity and criterion. The indirect effect of sensitivity, a_1_b_1_ = −.02, 95% CI [−.06, .01], was not significant. The indirect effect of criterion, a_2_b_2_ = .05, 95% CI [.003, .104], was significant. Note: ^*^p < .05, ^**^p < .01.

A multiple mediation model (see Figure 1) revealed a significant indirect effect; older adults exhibited a greater truth bias than young adults, which was associated with greater fraud, a_2_b_2_ = .05, 95% CI [.003, .104].

## Discussion

As predicted by Spreng et al. (2021), we found evidence that truth bias was a risk factor for fraud. A significant indirect effect indicated that older adults exhibited a greater truth bias than young adults when assessing the veracity of emotional pleas, and this was associated with larger donations to deceptive murderers. However, there was no direct relationship between age and fraud. Thus, while truth bias appears to be positively associated with fraud for older adults in highly emotional contexts, unmeasured protective factors affecting older adults’ financial decisions (e.g., social supports; Beach et al., 2018) or unmeasured risks affecting young adults (e.g., poor financial literacy; Xiao et al., 2014) may cancel out any direct effect of age on fraud. Findings dovetail with recent work by Bailey et al. (2018) which suggests that older (vs. young) adults experience greater empathy for those in pain, but do not differ in the extent to which they offer help to others. In other words, while older and young adults may differ in their evaluations or reactions to social stimuli, their offers of assistance (financial or otherwise) may not (see also Ross, Grossman & Schryer, 2014). In our study, both older and young adults donated similar, non-trivial amounts of money to deceptive murderers ($31).

While our findings provide insight into age-related differences, the cross-sectional nature of these findings cannot be assumed to reflect a causal effect of aging (Lindenberger et al., 2011). This concern is tempered to some extent by previous research which suggests that age-related differences in trust can be identified in longitudinal data, too (Poulin & Haase, 2015). However, future research should consider using longitudinal research methods. Additionally, while our stimuli improve upon the ecological validity of previous research by using real, high-stakes truths and lies with conceivable financial consequences, they differ from actual experiences of fraud. For example, participants had no opportunity to interact with the pleaders to ask questions and gather further information prior to making decisions about veracity or helping (Levine, 2018). Future research should consider whether these factors affect deception detection ability and, ultimately, fraud. Last, it should be acknowledged that the participants in the current study were a convenience sample of young and older adults, which may be atypical from the general population. Future research should replicate and extend these findings to other samples of older and young adults.

### Conclusion

While all participants evidenced a truth bias, older (v. young) adults were particularly biased to believe deceptive murderers, which was associated with greater monetary donations. However, donations did not differ by age, which may suggest other unmeasured protective factors for older adults or risk factors for young adults. Future research should adopt longitudinal methods to further understand multiple psychological mechanisms underlying fraud across the lifespan.

## Note

1 Several other questions and measures were gathered, which are not germane to the hypotheses tested here. Data from all measures can be found on OSF at: https://osf.io/64qut/

## References

[bibr1-07334648211049716] Bailey P. E. Brady B. Ebner N. C. Ruffman T. (2018). Effects of age on emotion regulation, emotional empathy, and prosocial behavior. The Journals of Gerontology: Series B, 75(4), 802–810. 10.1093/geronb/gby084. 30016531

[bibr2-07334648211049716] Beach S. R. Schulz R. Sneed R. (2018). Associations between social support, social networks, and financial exploitation in older adults. Journal of Applied Gerontology, 37(8), 990–1011. 10.1177/0733464816642584. 27255685 PMC7282700

[bibr17-07334648211049716] Borson S. Scanlan J. M. Chen P. Ganguli M. (2003). The Mini-Cog as a screen for dementia: Validation in a population-based sample. Journal of the American Geriatrics Society, 51(10), 1451-1454. 10.1046/j.1532-5415.2003.51465.x. 14511167

[bibr3-07334648211049716] Levine T. R. (2018). Ecological validity and deception detection research design. Current Molecular Medicine, 12(1), 45–54. 10.1080/19312458.2017.1411471.

[bibr4-07334648211049716] Li T. Fung H. H. (2012). Age differences in trust: An investigation across 38 countries. The Journals of Gerontology Series B: Psychological Sciences and Social Sciences, 68(3), 347–355. 10.1093/geronb/gbs072. 22929393

[bibr5-07334648211049716] Lindenberger U. Von Oertzen T. Ghisletta P. Hertzog C. (2011). Cross-sectional age variance extraction: What’s change got to do with it? Psychology and Aging, 26(1), 34–47. 10.1037/a0020525. 21417539

[bibr18-07334648211049716] Macmillan N. A. Kaplan H. L. (1985). Detection theory analysis of group data: Estimating sensitivity from average hit and false-alarm rates. Psychological Bulletin, 98(1), 185-199. 10.1037/0033-2909.98.1.185. 4034817

[bibr6-07334648211049716] Poulin M. J. Haase C. M. (2015). Growing to trust: Evidence that trust increases and sustains well-being across the life span. Social Psychological and Personality Science, 6(6), 614–621. 10.1177/1948550615574301.

[bibr7-07334648211049716] Ross M. Grossmann I. Schryer E. (2014). Contrary to psychological and popular opinion, there is no compelling evidence that older adults are disproportionately victimized by consumer fraud. Perspectives on Psychological Science, 9(4), 427–442. 10.1177/1745691614535935. 26173274

[bibr8-07334648211049716] Ruffman T. Murray J. Halberstadt J. Vater T. (2012). Age-related differences in deception. Psychology and Aging, 27(3), 543–549. 10.1037/a0023380. 21463058

[bibr16-07334648211049716] Spreng R. N. Ebner N. C. Levin B. E. Turner G. R. (2021) Aging and financial exploitation risk. In: Factora R.M. (eds) Aging and Money: Reducing risk of financial exploitation and protecting financial resources *(pp.55–73)*. Springer. 10.1007/978-3-030-67565-3_5

[bibr10-07334648211049716] Stanislaw H. Todorov N. (1999). Calculation of signal detection theory measures. Behavior Research Methods, Instruments, & Computers, 31(1), 137–149. 10.3758/bf03207704. 10495845

[bibr11-07334648211049716] Stanley J. T. Blanchard-Fields F. (2008). Challenges older adults face in detecting deceit: The role of emotion recognition. Psychology and Aging, 23(1), 24–32. 10.1037/0882-7974.23.1.24. 18361651

[bibr12-07334648211049716] ten Brinke L. Porter S. (2012). Cry me a river: Identifying the behavioral consequences of extremely high-stakes interpersonal deception. Law and Human Behavior, 36(6), 469–477. 10.1037/h0093929. 23205594

[bibr13-07334648211049716] TrueLink . (2019, April 6). The true link report on elder financial abuse 2015. http://documents.truelinkfinancial.com/True-Link-Report-On-Elder-Financial-Abuse-012815.pdf.

[bibr14-07334648211049716] Weissberger G. H. Goodman M. C. Mosqueda L. Schoen J. Nguyen A. L. Wilber K. H. Gassoumis Z. D. Nguyen C. P. Han S. D. (2019). Elder abuse characteristics based on calls to the national center on elder abuse resource line. Journal of Applied Gerontology, 39(10), 1078–1087. 10.1177/0733464819865685. 31364442 PMC6992470

[bibr15-07334648211049716] Xiao J. J. Ahn S. Y. Serido J. Shim S. (2014). Earlier financial literacy and later financial behaviour of college students. International Journal of Consumer Studies, 38(6), 593–601. 10.1111/ijcs.12122.

